# Hyperglycemia Alters the Structure and Hemodynamics of the Developing Embryonic Heart

**DOI:** 10.3390/jcdd5010013

**Published:** 2018-02-12

**Authors:** Taylor B. Lawson, Devon E. Scott-Drechsel, Venkat Keshav Chivukula, Sandra Rugonyi, Kent L. Thornburg, Monica T. Hinds

**Affiliations:** 1 Biomedical Engineering Department, Oregon Health & Science University, Portland, OR 97239, USA; devonscottster@gmail.com (D.E.S.-D.); keshav008@gmail.com (V.K.C.); rugonyis@ohsu.edu (S.R.); hindsm@ohsu.edu (M.T.H.); 2 Knight Cardiovascular Institute, Oregon Health & Science University, Portland, OR 97239, USA; thornbur@ohsu.edu

**Keywords:** diabetes, development, congenital heart defect, hemodynamics

## Abstract

Congenital heart defects (CHDs) represent the most common form of human birth defects; approximately one-third of heart defects involve malformations of the outflow tract (OFT). Maternal diabetes increases the risk of CHD by 3–5 fold. During heart organogenesis, little is known about the effects of hyperglycemia on hemodynamics, which are critical to normal heart development. Heart development prior to septation in the chick embryo was studied under hyperglycemic conditions. Sustained hyperglycemic conditions were induced, raising the average plasma glucose concentration from 70 mg/dL to 180 mg/dL, akin to the fasting plasma glucose of a patient with diabetes. The OFTs were assessed for structural and hemodynamic alterations using optical coherence tomography (OCT), confocal microscopy, and microcomputed tomography. In hyperglycemic embryos, the endocardial cushions of the proximal OFT were asymmetric, and the OFTs curvature and torsion were significantly altered. The blood flow velocity through the OFT of hyperglycemic embryos was significantly decreased, including flow reversal in 30% of the cardiac cycle. Thus, hyperglycemia at the onset of gestation results in asymmetric proximal endocardial cushions, abnormal OFT curvature, and altered hemodynamics in the developing heart. If present in humans, these results may identify early developmental alterations that contribute to the increased risk for cardiac malformations in babies from diabetic mothers.

## 1. Introduction

The incidence of diabetes is rising worldwide. Maternal diabetes increases the risk of congenital cardiac malformations by 3–5 fold [1] with malformations induced before the seventh week of gestation [2,3,4], when the heart undergoes organogenesis. This increased risk of malformations is the result of maternal glucose freely crossing the placenta by facilitated diffusion and entering the embryonic circulation. The risk for a malformed heart is determined by the degree of hyperglycemia during cardiac organogenesis (higher risk with less glycemic control) [4]. It is critical to understand the effects of hyperglycemia on heart development in the embryo during organogenesis. Changes in cardiac structure, in turn, affect blood flow conditions. Heart development is known to be dependent on blood flow [5,6,7], and abnormal embryonic blood flow conditions lead to a spectrum of detrimental cardiovascular alterations and cardiac defects [8,9]. However, little is known about the effect of hyperglycemia on embryonic cardiac structures or on the resulting changes in hemodynamics. In this study, we determine the changes in the structure and hemodynamic conditions that hyperglycemia induces within the developing looped heart.

Chick models are used to study the early stages of embryonic heart development because of the many advantages of the embryonic chick. Importantly, developmental processes, particularly heart development, are highly conserved among vertebrate species and, therefore, results from the chick often apply to humans. Further, the chick embryo is easy to access; the heart can be easily observed and analyzed without affecting cardiac growth. Our team has extensive experience working with chick embryos and has developed technologies to image the beating chick heart simultaneously with the hemodynamic conditions in vivo [10,11,12,13,14,15,16]. In early developmental stages, mammalian and chick hearts have the same structure and lack an insulin-sensitive transporter [17,18,19]. At Hamburger–Hamilton stage 18 (HH18), corresponding to day 3 of chick development, the chick heart has a tubular shape with primitive valves and consists of the atrium, the ventricle, and the outflow tract (OFT). The OFT connects the primitive ventricle to the arterial system. Within the OFT, localized protrusions of cardiac jelly form the endocardial cushions which function as the primitive valves; two sets of these protrusions exist, located proximally and distally within the OFT. Our studies herein focus on the proximal set of cushions. We direct our focus in this study to the OFT because during early stages of development, the OFT is sensitive to changes in environmental conditions including glycemic and hemodynamic stresses [20,21]. Subsequently, the OFT undergoes extensive morphogenesis to develop into the semilunar valves and the aortic and pulmonary trunks. Further, the OFT provides a specific cardiac segment to study, in which we can simultaneously measure the structure and hemodynamic stresses. Unlike mammalian models of embryonic development, the chick model allows us to control the precise timing and the extent of hyperglycemia and does not include other influences from the mother, enabling the identification of alterations due to hyperglycemia alone. 

This study was designed to test the hypothesis that continuous hyperglycemia in the avian embryo would alter cardiac development and lead to abnormal heart structures, which would in turn have significant effects on the in vivo blood flow through the heart. To test this hypothesis, a chicken model of embryonic heart development was monitored during a period of continuous elevated blood glucose [22] from the onset of incubation through the formation of the looped heart. The quantification of both the OFT structure and blood flow was performed *in ovo* using optical coherence tomography (OCT). The changes in the OFT structure were confirmed and quantified at a later stage using confocal microscopy and microcomputed tomography (micro-CT).

## 2. Results

### 2.1. Hyperglycemia Causes Asymmetric Endocardial Cushions

**Figure 1:**
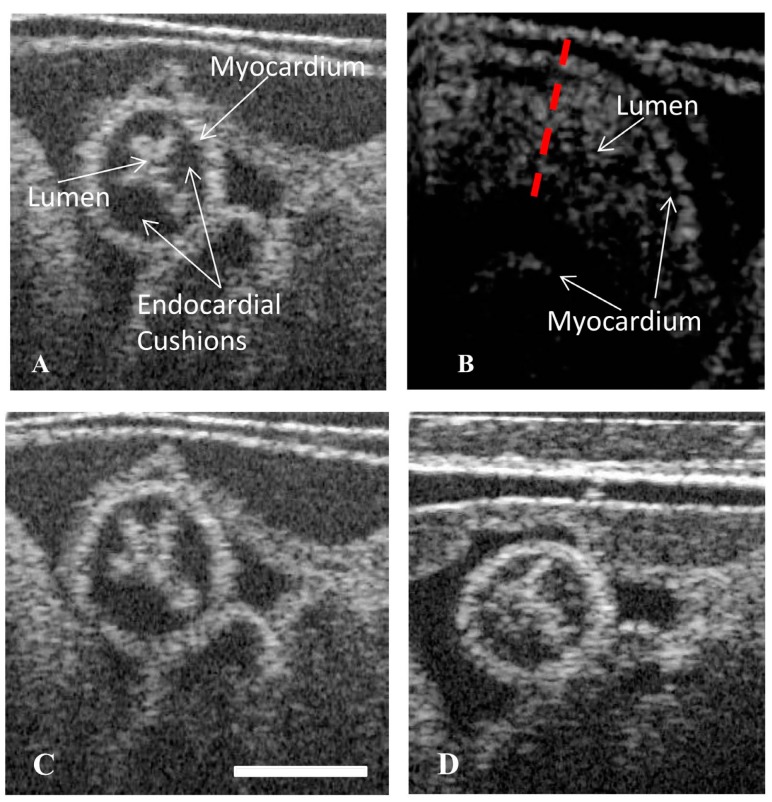
Hyperglycemia can lead to asymmetric endocardial outflow tract (OFT) cushions visible in the displaced lumen of cross-sectional images. Cross-sectional optical coherence tomography (OCT) images of the OFT showing a vehicle control (**A**), a vehicle control cross section labelled for reference (**B**), an osmotic control (**C**), a d-glucose-treated embryo with the lumen displaced towards the ventral portion of the OFT (**D**). Scale bar = 70 µm.

OCT imaging of live chicken embryos at stage HH18 provided real-time images of the beating embryonic OFT. From these images, the cross-sectional views of the OFT were extracted, enabling the visualization of the microstructure. In these views, the myocardium, lumen, and the endocardial cushions were discernable. The hyperglycemic embryos had a higher occurrence of lumens that were displaced to the ventral side of the OFT. Concomitant with the off-centered lumen, the inferior OFT cushion was larger than the superior cushion, thereby causing the lumen to be pushed superiorly. In both the control embryos (vehicle and l-glucose), the lumen was positioned in the center of the OFT (Figure 1A,B). Asymmetric, proximal cushions with the lumen positioned ventrally (Figure 1D) were found in six embryos exposed to d-glucose, compared to one exposed to l-glucose. None of the vehicle control embryos were affected (Table 1). 

**Figure 2:**
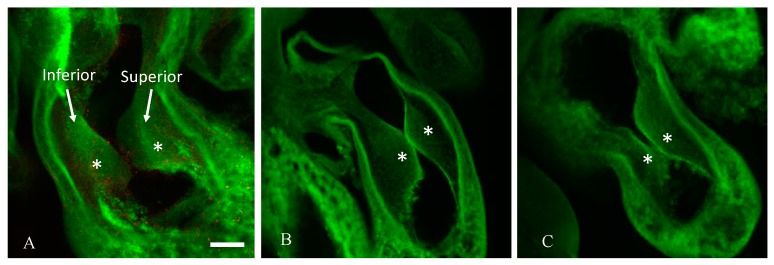
Hyperglycemia induces asymmetric endocardial OFT cushions at Hamburger–Hamilton stage 24 (HH24). Representative confocal images of a slice of the OFT cushions taken in the sagittal plane cushions labeled, and asterisks marking the two endocardial cushions from a representative vehicle control (**A**), an osmotic control (**B**), and a d-glucose-treated embryo (**C**). Scale bar = 100 µm, equivalent across images.

**Figure 3:**
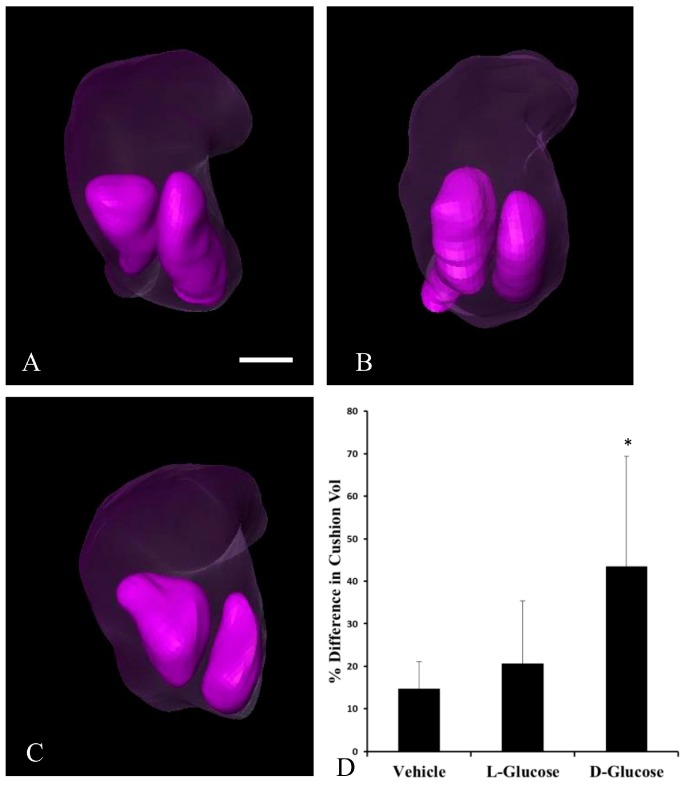
Segmented HH24 OFTs indicate endocardial OFT cushion asymmetry in response to hyperglycemia. Representative, reconstructed microcomputed tomography (Micro-CT) images of the OFT (transparent purple) with the cushions, left inferior, right superior, (purple) identified for a vehicle control (**A**), an osmotic control (**B**), and a d-glucose-treated embryo (**C**). (**D**) Average percentage difference in OFT cushion volume. Scale bar = 100 µm, equivalent across images. * *p* < 0.05 ANOVA.

To determine the degree to which cushion asymmetry persisted during development, the differences in cushion volume were quantified in HH24 embryos. By analyzing the cushion volume, the percent difference in the volume of one cushion compared to the other cushion for each embryo was calculated. The analysis of the confocal images (Figure 2) indicated that d-glucose embryos displayed a higher than average percent difference in cushion volume compared to l-glucose and vehicle (data not shown). To confirm this asymmetry in HH24 embryos, micro-CT imaging was used (Figure 3). There were no significant differences between treatments in the surface area and volumes of the atria and ventricle. However, in the OFT, the d-glucose-treated embryos had a significantly lower surface area and volume compared to the vehicle and osmotic control. The volume of each cushion was measured, and the percent difference in OFT cushion size was calculated for every embryo. Similar to the confocal microscope measurements, the d-glucose embryos showed a higher percent difference in cushion size compared to the two control conditions (Figure 3D). 

### 2.2. Hyperglycemia Alters the Curvature of the OFT

**Figure 4:**
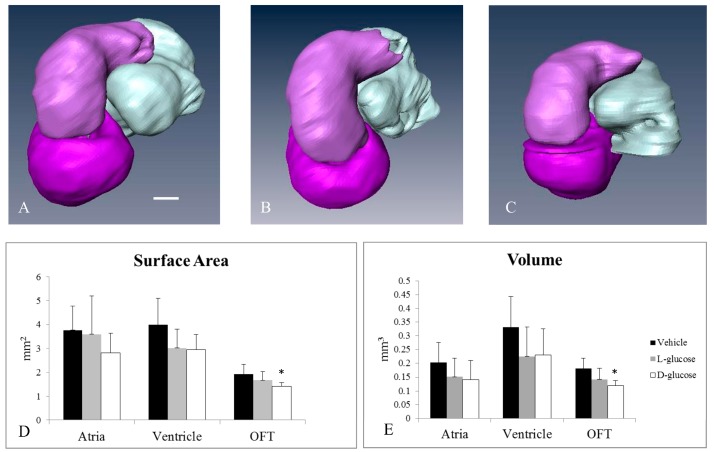
Micro-CT analysis of HH24 hearts reveals differences in endocardial OFT properties in response to hyperglycemia. Representative, reconstructed micro-CT images of the atria (white), ventricle (dark purple), and OFT (light purple) for a vehicle control (**A**), an osmotic control (**B**), and a d-glucose-treated embryo (**C**). The average surface area (**D**) and volume (**E**) for the heart sections. Scale bar = 100 µm. * *p* < 0.05 ANOVA.

**Figure 5:**
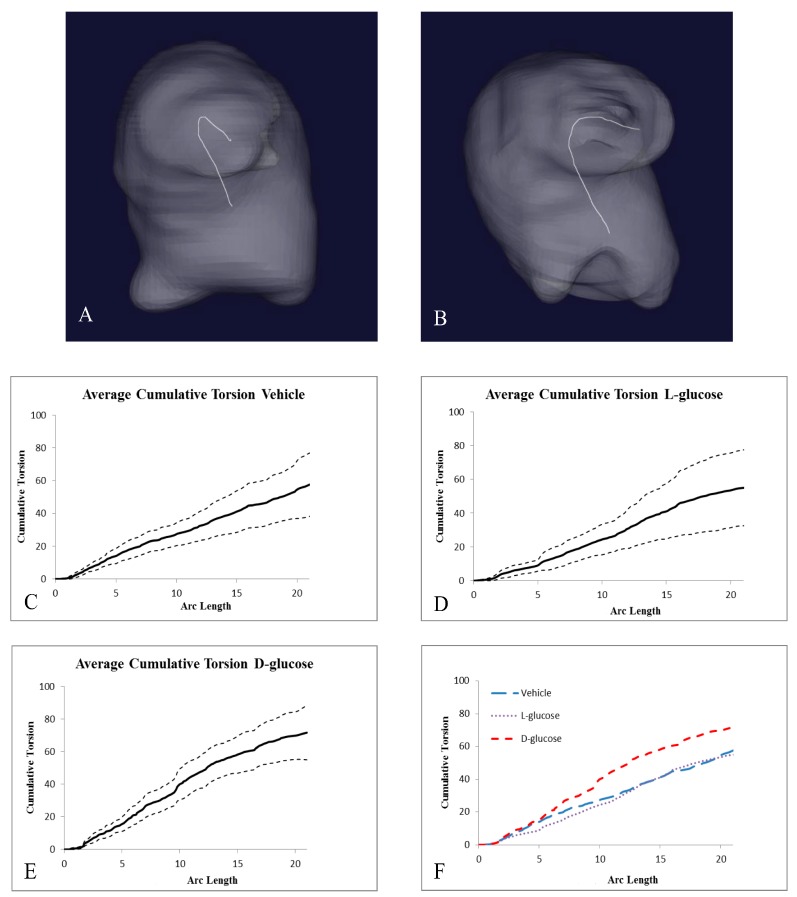
Hyperglycemic conditions can result in changes in the torsion of the OFT. 3D centerlines of a HH24 representative (**A**) vehicle control embryo and (**B**) d-glucose-treated embryo to quantify the amount of torsion of the OFT. (**C**–**E**) Graphs, separated by treatment, of the average cumulative torsion, with the dashed lines representing + and −1 standard deviations of the OFT torsion plotted against the cumulative arc length. (**F**) The average cumulative torsion of the three treatments plotted against the average arc length.

The analysis of micro-CT images indicated the structural differences caused by hyperglycemia (Figure 4). The surface areas and volumes of the atria and ventricles of the d-glucose, l-glucose, and vehicle control embryos were not significantly different among the groups. Yet, both the surface area and volume of the OFT of the d-glucose-treated embryos were significantly smaller compared the l-glucose and vehicle control embryos. Additional analyses of the OFT calculated the torsion for the midline of the OFT using VMTK and MATLAB computations. The average cumulative torsion for each treatment, d-glucose (*n* = 9), l-glucose (*n* = 9), and vehicle (*n* = 12), were plotted against the average arc length (Figure 5). The cumulative torsion for d-glucose-treated embryos was significantly greater than that of either the osmotic control or the vehicle control (Table 2).

### 2.3. Hyperglycemia Reduces Altered Blood Flow Velocity in the OFT

**Figure 6:**
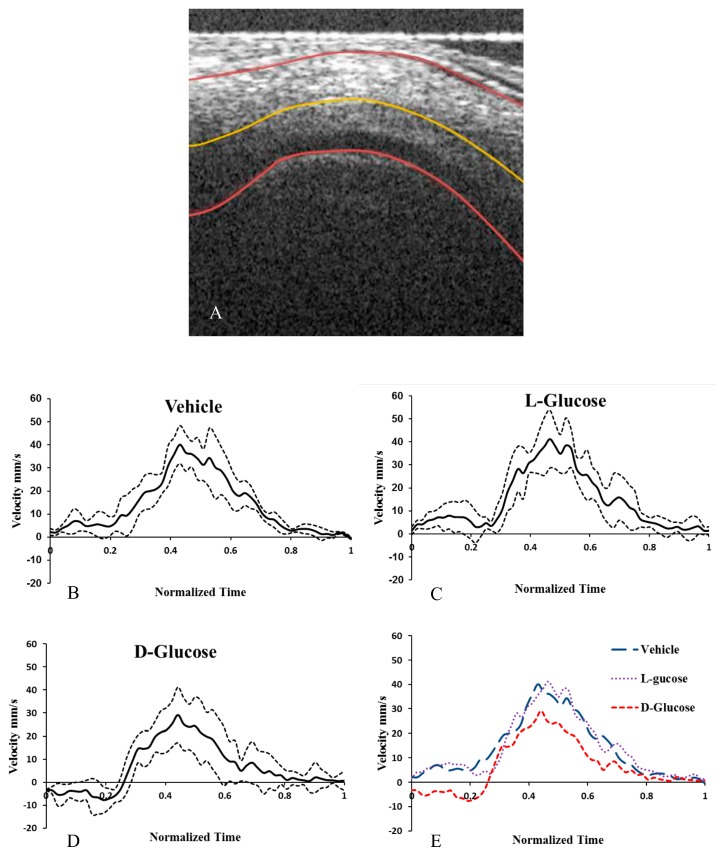
Hyperglycemia alters the hemodynamics in chick embryonic OFT. OCT image of the longitudinal cross section of a vehicle control HH18 OFT with lumen and myocardium labelled (**A**). The outer walls and centerline of the OFT identified with the custom MATLAB program. The calculated centerline velocity of the vehicle control (**B**), osmotic control (**C**), and d-glucose-treated embryos (**D**) from the Doppler OCT images. The average velocities (**E**) for the treatments. Scale bar = 70 µm. * *p* < 0.05 ANOVA.

From OCT Doppler data, the average blood flow velocity along the midline of the HH18 OFT was determined for the cardiac cycle. In the d-glucose-injected hyperglycemic embryos, there was backflow at the beginning of the cycle that was not present in the two control conditions (Figure 6). Consequently, in the d-glucose embryos the average velocity and minimum velocity were significantly lower and, importantly, the percent of the cardiac cycle with back flow was significantly higher than the in vehicle and the l-glucose embryos (Table 3).

## 3. Discussion

Maternal hyperglycemia has been shown to increase the risk of congenital heart defects by as much as 3–5 fold. Cardiac defects commonly found in offspring of diabetic mothers include ventricular septal defects, transposition of great arteries, and aortic stenosis [23]. This study takes a step toward understanding the origin of these defects and the mechanisms by which they develop. The primary findings of the study are that the hyperglycemic stress causes persisting endocardial OFT cushion asymmetry, alteration of the curvature of the OFT, and reversal of the blood flow through the OFT at early embryonic stages. These early structural and hemodynamic changes are likely to lead to the cardiac defects seen in the offspring of diabetic mothers. 

The proper symmetric formation of the endocardial cushions is critical to normal heart development because these cushions develop into cardiac valves, as well as atrial and membranous ventricular septa [24]. As the OFT develops, the endocardial cushions grow towards one another, finally fusing in order to form a continuous septum. This study provides evidence for the impact of hyperglycemia on endocardial cushion development in the chick embryo at stages HH18 and HH24. At stage HH18, the hyperglycemic embryos presented with a ventrally displaced lumen. This was hypothesized to be the result of one endocardial cushion being larger than the other. Confocal microscopy and micro-CT imaging of the heart and OFT cushions in HH24 embryos confirmed the presence of asymmetric cushion sizes in the hyperglycemic embryos. Previous studies have found a hypoplastic myocardium and endocardial cushions present in mice embryos, E9.5, 10.5, and 11.5 (equal to stages HH 14, 17, 19 in chick embryos) from mothers with streptozotocin-induced diabetes. Those embryos from diabetic mothers had inhibited cell proliferation with unaltered apoptosis in the developing endocardial cushions of both the OFT and atrioventricular (AV) junction of the embryonic heart [25], in conjunction with a decrease in mitosis, which thereby altered the structure of the cushions [25]. Similarly, decreased proliferation of the endocardial and myocardial cells was seen in our sustained hyperglycemic chick model [22]. The decreased proliferation was a result of the arrest of embryonic chick cardiac cells in the G_1_ phase of the cell cycle due to the down-regulation of cyclin D1 and the upregulation of p21 [22]. Thus, the asymmetrical endocardial cushions may be due to altered cellular proliferation of the cells within the endocardial OFT cushions as a result of hyperglycemia altering cell cycle gene expression. 

The hyperglycemic conditions in this study likely altered both the neural crest cells and the cardiac cells responsible for the structure of the developing OFT. The embryonic heart and OFT develop from a variety of cells including endocardial, myocardial, and neural crest cells. Prior research of cells under high glucose concentrations, cultured in 50 mM d-glucose or derived from mouse embryos with streptozotocin-induced diabetes, illustrated a depression in the proliferation of cardiac neural crest cells [26,27]. Beginning early in embryonic development, at stage HH11 in the chick embryo, neural crest cells migrate from the neural crest to the dorsal region of the neural tube, which leads to the formation of the OFT [28]. Morgan et al. demonstrated that hyperglycemia and oxidative stress on E7.5 mouse embryos (equal to HH11 in chick embryos) disrupted cardiac neural crest cell migration and led to cardiac OFT defects [29]. Oxidative stress was a result of the hyperglycemia and led to the inhibition of the expression of genes vital for cardiac neural crest cell viability. This deleterious effect on cardiac neural crest cells resulting from hyperglycemia and oxidative stress caused apoptosis of the cardiac neural crest cells during migration to the OFT and subsequent OFT defects [29]. Others have also shown a decrease in endothelial cell proliferation as a result of hyperglycemia and hyperosmolarity. Larger et al. carried out in vivo studies examining the impact of hyperglycemia on angiogenesis. Results of the study indicated that hyperglycemia decreased the rate of proliferation and induced apoptosis of endothelial cells [30]. In conjunction with the known impaired proliferation and migration of cardiac neural crest cells, research by Scott et al. found a decrease in endocardial and myocardial cellular proliferation in the OFT as a result of alterations in regulators of the cell cycle, specifically an increase in p21 and a decrease in cyclin D1 [22]. Thus, the decrease in proliferation of cardiac neural crest cells, vital to OFT development, along with a decrease in the proliferation of endocardial cells within the developing OFT, both in response to hyperglycemia, appear to play a role in the observed development of structural malformations. The OFT is formed from a complex interplay of processes involving several different cell types, including the migration and differentiation of neural crest and secondary heart field cells, as well as the proliferation of their progeny. Normally, the secondary heart field cells equally populate both OFT cushions. The asymmetry of the proximal OFT cushions due to hyperglycemia likely results from a combination of reduced or altered cellular distribution of the cells within the cushions and a curved OFT. The latter exacerbates the asymmetry because of the altered hemodynamics affecting one cushion more than the other. A change in hemodynamics causes different amounts of glucose transported to the OFT walls and changes the pressure, strain, and wall shear stress experienced by each cushion [11,31]. This differential transport could impact the migration and differentiation of the neural crest and secondary heart field cells, as well as the proliferation of the differentiated cardiac cells.

Endocardial cells arise from distinct endothelial lineages, line the walls of the heart, and are particularly sensitive to hemodynamic conditions. Endothelial cells act as force sensors and transducers, responding to varying levels of wall shear stress, and are greatly influenced by the blood flow and the forces it exerts on the walls of the heart [32]. The response of endothelial cells depends on both the magnitude and the direction of the applied shear stress, indicating the vital role hemodynamics may hold in the development of the valves and vessels. Previous *in vivo* studies have shown that mechanical interference with flow streamlines within the chick embryonic heart resulted in a variety of heart defects including OFT abnormalities [32]. Furthermore, the wall shear stress within the OFT in a stage-HH18 embryo has been shown to vary spatially and temporally, with the distal portion of the OFT wall experiencing larger wall stresses compared to the proximal portion of the OFT [33]. This uneven distribution of stresses and strains is likely compounded by the asymmetric endocardial cushions in response to hyperglycemia. This compounding effect likely alters biomechanical cues to endocardial cells, affecting the normal differential growth and remodeling patterns during cardiac development. Surgical interventions that altered the blood flow indicated that the types of defects are dependent on the degree of hemodynamic changes, including those most commonly witnessed clinically, such as ventricular septal defects, pharyngeal arch artery malformations, and double outlet right ventricle [31]. This provides further evidence that hemodynamics play a crucial role in the formation of cardiac malformations. Identifying the separate effects of hemodynamics and other factors such as teratogen exposure remains to be performed and likely can only be done with either temporal exposure to hyperglycemia or cell-specific hyperglycemic conditions. 

In summary, hyperglycemia, as a result of the diabetic condition, disturbs normal embryonic heart development. Alterations in cellular proliferation in the endocardial cushions likely contribute to a structural difference between the two cushions in hyperglycemic embryos. Malformations in the endocardial cushions during critical stages of heart development contribute to further structural alterations in the OFT, where the endocardial cushions are located. Structural malformations in the endocardial cushions and OFT, as a result of the teratogenic effect of hyperglycemia, alter blood flow patterns, which may further enhance the teratogenic effects of hyperglycemia and contribute to secondary malformations in the vasculature. These results identify early developmental alterations that contribute to the increased risk for cardiac malformations in babies from diabetic mothers.

## 4. Materials and Methods

### 4.1. Embryo Hyperglycemia

To induce a sustained elevation in plasma glucose, glucose was injected directly into the yolk of fertilized white leghorn chicken eggs. At day 0, before incubation, a 2 cm window was made in the air sac of the egg. Using a 30 g needle, 600 µL of 750 mM d-glucose was directly injected into the yolk, avoiding damage to the blastocyst. Controls were performed using chick saline (vehicle) and l-glucose (osmotic control). Our prior studies have validated the elevation of plasma glucose using this method. The injection of 750 mM d-glucose raised the plasma glucose concentration in HH24 embryos to 177 ± 20 mg/dL, a clinically significant level compared with 72 ± 9 mg/dL in the stage-matched unopened chick embryos [34]. A clinical diagnosis of diabetes comes with a fasting plasma glucose level of 126 mg/dL and greater [35]. l-glucose raises the circulating level of glucose in the plasma, but is not actively transported into the cells and therefore should not affect cell functions. The window was sealed with plastic wrap, and the eggs incubated at 38 °C. The eggs were incubated to stage HH18 (~3 days) or to stage HH24 (~4 days). d-glucose embryos were significantly developmentally delayed [34], therefore they needed longer incubation times. For HH18 embryos, d-glucose-injected eggs were incubated for 5–6 h longer than the eggs of control embryos to allow them to reach the correct stage. An additional period of 12 h, compared to control embryos, was required for the d-glucose eggs to develop to stage HH24. For all embryos, before embryonic isolation, blood glucose levels were quantified with a OneTouch Ultra Link Glucometer. HH18 embryos were used for optical coherence tomography (OCT) analysis, and HH24 embryos were utilized for confocal and microcomputed tomography (micro-CT) analyses. 

### 4.2. Dynamic Imaging of the OFT with Optical Coherence Tomography

OCT allows the *in vivo* visualization of intact embryos and cardiac dynamics at HH18. OCT is a noncontact and noninvasive imaging method that enables the visualization of microstructures and the calculation of blood flow velocities. The OCT system setup has been reported elsewhere [36]. Briefly, a spectral-domain OCT system was used to image the structure and blood flow of the chick OFT. The system used a superluminescent diode broadband light sources that had a full-width-half maximum of 56 nm centered at 1310 nm. The OCT had an axial spatial resolution of 10 µm and a lateral spatial resolution of 16 µm. A 1024-element infrared in Gas As line scan camera with a 14-bit digital depth and maximum line scan rate of 92 kHz was used to allow the acquisition of 2D images (B-mode) of 512 × 512 (512 A scans) at 140 frames per second. HH18 embryos were kept in their eggs while OFT cross-sectional and longitudinal images were taken. The cross-sectional and longitudinal images were positioned at the center of the chick OFT.

The blood flow velocities of stage HH18 embryonic hearts were quantified using 2D Doppler OCT imaging and reconstruction procedures. The Doppler OCT system imaged the longitudinal section of the OFT of each embryo and generated flow phase images via established post-processing calculations [36]. Briefly, from the extracted structural and Doppler phase-shift image datasets of the OCT raw data, a custom MATLAB program was used to create images of the blood flow and OFT wall movement [36]. The flow angle was then measured using a custom MATLAB program by first outlining the upper and lower myocardium walls at maximal expansion in 2D structural images and then calculating the tangent to the OFT centerline at maximum expansion [36]. The structural outlines of the myocardium and centerline were then overlaid on corresponding Doppler flow images from which the velocity measurement position along the centerline was selected [36]. Finally, at the measurement location, the angle of the line tangent to the centerline was used to calculate the velocity [36]. A blinded observer viewed and quantified the number of incidences of asymmetric cushions in OCT images from a total of 10 embryos per treatment (total of 30 embryos).

### 4.3. Static Structural Imaging of the OFT

The embryonic cushion volume and symmetry were determined in both HH18 and HH24 embryos. Whole HH24 embryo samples were fixed overnight, dehydrated, and placed in clearing solution. The natural autofluorescence of the embryonic tissue enabled the analysis of the heart structure without the use of exogenous fluorophores in the HH24 embryos. The OFTs from HH24 embryos were imaged using a confocal laser-scanning microscopy (Bio-Rad MRC500 on a Zeiss Axiovert inverted microscope). Imaged data was collected in 3D as a Z-series of ~10 μm/steps. Z-series were analyzed using ImageJ (NIH) software, and the reconstructed 3D maps of the OFT revealed cushion alterations. The endocardial cushion volumes were found by measuring the volume of each slice of cushion through the entire volume of the cushions in ImageJ software. Each cushion was manually outlined using ImageJ; the surface area was calculated and multiplied by the step thickness to calculate the volume within the slice. The total OFT volume was the summation of the slice volumes for each image in the cushion z stack (between 11 and 20 slices per OFT). 

Micro-CT imaging provided information about the overall structure and size of the embryonic heart. Similar to confocal microscopy, micro-CT analysis allowed the visualization of embryos at a later stage, compared to OCT, to quantify the structural differences seen at HH24. Micro-CT imaging was utilized because of the absence of embryo manipulation and dehydration that is required in confocal microscopy. At stage HH24, the embryos were removed from the eggs and placed in PBS, while the membranes surrounding the embryos were removed. The embryos were then immediately transferred to an iodine and potassium iodide contrast agent solution, Lugol’s Solution (Sigma) and soaked in Lugol’s Solution for 24 h. A Caliper Quantum FX micro-CT was used to take 20 μm resolution images of the embryos. The generated images were analyzed using Amira software, and 3D volume-rendering reconstructions of the hearts were created. The quantification of the volumes and surface areas of the ventricle, the atria, the OFT and the cushions was performed, and changes in the curvature of the OFT and looping of the heart were determined.

### 4.4. Structural Analysis

Using Amira software to analyze the 360° images from micro-CT scans, the external sections of the heart, including the atria, ventricle OFT, and each OFT cushion, were segmented. The surface area and volumes of the atria, ventricle, and OFT were compared among treatments. From the manual segmentation Amira, 3D volume renderings of the structures of the heart were generated, and surface areas and volumes calculated. Additionally, the analysis of the 3D volume rendering of embryonic OFTs was performed using the Vascular Modeling Toolkit (VMTK) [37]. VMTK, a toolkit for 3D reconstruction, geometric analysis, and mesh generation of blood vessels, allowed for the quantification of the structural changes in the embryonic OFT based on acquired micro-CT images processed using Amira software. To quantify the structural changes, the torsion of the centerlines for each OFT was computed with VMTK based on the outer myocardial walls. Within VMTK, the inlet, at the transition point from ventricle to OFT, and outlet, at the transition point from OFT to atrium, on the 3D reconstructed OFT were user-specified. Then, VMTK extrapolated a 3D centerline curve of the OFT, generating Cartesian coordinates, and the values of curvature and torsion at each point along the curve. The curvature is the magnitude of the second derivative of the curve at that point. The torsion of a curve measures how sharply it is twisting out of the plane of curvature. The specified inlet and outlet points were maintained consistent at the specific transition points between individual OFTs. A custom MATLAB program was utilized to compute the cumulative torsion and arc length along each OFT. The cumulative torsion was then plotted against the cumulative arc length for each OFT, and averages were computed for each treatment.

### 4.5. Statistical Analyses

Statistical analyses were performed using SPSS, v. 22. For all analyses, an ANOVA was performed to compare the three treatments: vehicle control, l-glucose osmotic control, and d-glucose. A Tukey post-hoc analysis was done to identify the significantly different treatments.

## Figures and Tables

**Table 1:** Incidence of OCT Cushion Asymmetry.

Treatment	Total No. Embryos	Cushion Asymmetry
**Vehicle**	10	0
**l-glucose**	10	1
**d-glucose**	10	6 *

ANOVA, * *p* < 0.05 compared to vehicle and l-glucose.

**Table 2:** Cumulative Torsion Statistical Analysis.

Treatment	Number of Samples	Cumulative Torsion	Average Arc Length
**Vehicle**	12	57.01 ± 19.19	20.84 ± 6.92
**l-Glucose**	9	55.06 ± 22.58	21.15 ± 5.93
**d-Glucose**	8	71.27 ± 16.09 *^+^	20.75 ± 8.30

ANOVA, *^+^ *p* < 0.05.

**Table 3:** Hemodynamic parameters for each treatment.

Treatment	Average Velocity (mm/s)	Average Peak Velocity (mm/s)	Average Min. Velocity (mm/s)	% of Cycle with Back Flow	Cardiac Period (s)
**Control**	13.9 ± 3.8	40.7 ± 12.4	−0.60 ± 0.79	6.3 ± 5.2	0.39 ± 0.02
**l-Glucose**	17.4 ± 5.2	41.5 ± 15.6	−1.73 ± 3.8	2.4 ± 3.7	0.406 ± 0.015
**d-Glucose**	8.0 ± 3.1*^+^	31.4 ± 8.2	−12.4 ± 6.9 *^+^	30.1 ± 10.3*	0.413 ± 0.023

ANOVA, *^+^ *p* < 0.05.
